# A *Drosophila* Mitochondrial Complex I Deficiency Phenotype Array

**DOI:** 10.3389/fgene.2019.00245

**Published:** 2019-03-27

**Authors:** Sarah Foriel, G. Herma Renkema, Yvonne Lasarzewski, Job Berkhout, Richard J. Rodenburg, Jan A. M. Smeitink, Julien Beyrath, Annette Schenck

**Affiliations:** ^1^Khondrion B.V., Nijmegen, Netherlands; ^2^Radboud Center for Mitochondrial Medicine, Department of Pediatrics, Radboud University Medical Center, Nijmegen, Netherlands; ^3^Department of Human Genetics, Donders Institute for Brain, Cognition and Behaviour, Radboud University Medical Center, Nijmegen, Netherlands

**Keywords:** mitochondrial disease, complex I deficiency, *Drosophila melanogaster*, disease model, screening

## Abstract

Mitochondrial diseases are a group of rare life-threatening diseases often caused by defects in the oxidative phosphorylation system. No effective treatment is available for these disorders. Therapeutic development is hampered by the high heterogeneity in genetic, biochemical, and clinical spectra of mitochondrial diseases and by limited preclinical resources to screen and identify effective treatment candidates. Alternative models of the pathology are essential to better understand mitochondrial diseases and to accelerate the development of new therapeutics. The fruit fly *Drosophila melanogaster* is a cost- and time-efficient model that can recapitulate a wide range of phenotypes observed in patients suffering from mitochondrial disorders. We targeted three important subunits of complex I of the mitochondrial oxidative phosphorylation system with the flexible UAS-Gal4 system and RNA interference (RNAi): NDUFS4 (ND-18), NDUFS7 (ND-20), and NDUFV1 (ND-51). Using two ubiquitous driver lines at two temperatures, we established a collection of phenotypes relevant to complex I deficiencies. Our data offer models and phenotypes with different levels of severity that can be used for future therapeutic screenings. These include qualitative phenotypes that are amenable to high-throughput drug screening and quantitative phenotypes that require more resources but are likely to have increased potential and sensitivity to show modulation by drug treatment.

## Introduction

With one out of 5000 live births affected (Skladal et al., 2003; Schaefer et al., 2004), mitochondrial diseases are classified as rare diseases. They often are caused by defects of the oxidative phosphorylation (OxPhos) system (Smeitink et al., 2001). Mitochondrial diseases with early onset presentation show a broad phenotypic spectrum and are associated with poor prognosis and/or premature death in the majority of cases. Currently, only supportive care can be offered to patients. There is an urgent medical need for effective drugs to treat mitochondrial diseases to prevent their severe progression. Because mitochondrial diseases form a family of highly heterogeneous conditions (Distelmaier et al., 2009; Vafai and Mootha, 2012; Moggio et al., 2014), developing various animal models to understand the pathomechanisms of the different mitochondrial diseases and test treatment strategies is highly relevant (Foriel et al., 2015). The identification and the use of multiple models recapitulating a spectrum of symptoms encountered in patients with mitochondrial diseases may be more predictive and present a better strategy toward identification of effective therapeutics.

A great advantage of *Drosophila melanogaster* as a model organism, apart from its general features (Jennings, 2011; Pandey and Nichols, 2011), is the flexibility of the utilized UAS-Gal4 system to control the knockdown (KD) of specific genes (Brand and Perrimon, 1993). The yeast transcription factor Gal4 determines the spatial and temporal expression of a transgene located downstream of an Upstream Activating Sequence (UAS). The temperature sensitivity of this binary system (Duffy, 2002) allows to fine-tune the severity of the induced KD and therefore to adjust and select suitable conditions. We developed an array of KD models and phenotypes recapitulating several features of mitochondrial complex I deficiency as the most frequently mutated OxPhos complex (Loeffen et al., 2000; Smeitink et al., 2001; Fassone and Rahman, 2012). Among the 44 subunits forming the human CI, 42 are evolutionary conserved in *Drosophila*, highlighting the important role of each of these building blocks (Garcia et al., 2017). In this study, we used RNA interference (RNAi) to target the expression of three subunits known to play crucial roles in CI function and to be prone to mutations in patients (Zeviani et al., 2003; Koene et al., 2012; Lake et al., 2015; Mayr et al., 2015; Koopman et al., 2016). NDUFV1 (V1) and NDUFS7 (S7) are two core subunits that play crucial roles in the catalytic function of the complex: V1 is involved in the oxidation of NADH into NAD+, creating the flow of electrons. S7 is required for the transfer of electrons to ubiquinone. NDUFS4 (S4) on the other hand is an accessory subunit which appears to be important for the structural integrity of the complex (Janssen et al., 2006; Mimaki et al., 2012; Valsecchi et al., 2012; Varghese et al., 2015). Mutations in these subunits lead to very severe early onset symptoms and poor prognosis for the patients. For each subunit, we used two ubiquitously expressed Gal4 lines to induce the KD [actin-Gal4 and daughterless (da)-Gal4] and two temperatures (25 and 28°C) to generate a panel of phenotypes. We then based our screening on four parameters: the percentage of residual gene expression, the OxPhos complexes activity, the eclosion rate, and survival analysis. Here we report robust and disease-relevant phenotypes that could be suitable for future modification by pharmacologic treatment.

## Materials and Methods

### Utilized Stocks and Fly Husbandry

The KD of mitochondrial CI subunits was induced using the binary UAS-Gal4 system (Brand and Perrimon, 1993). The utilized RNAi lines were vdrc101489 to target ND-18 (the NDUFS4 *Drosophila* ortholog), vdrc110881 to target ND-20 (the NDUFS7 *Drosophila* ortholog), vdrc43184 to target ND-51 (the NDUFV1 *Drosophila* ortholog) and their genetic background vdrc60000 (control GD for NDUFV1) and vdrc60100 (control KK for NDUFS4 and NDUFS7) from Vienna *Drosophila* Resource Center (VDRC). In terms of sequence-related off-targets, these lines fulfill the highest standard: vdrc101489 (CG12203; NDUFS4) s19 = 1; off targets = 0; vdrc101881 (CG9172; NDUFS7) s19 = 1; off targets = 1, corresponding to CG2014, the second S7 paralog in *Drosophila* that is specifically expressed only in testis; vdrc43184 (CG9140; NDUFV1) s19 = 1; off targets = 0. In addition, both vdrc101489 and vdrc110881, the two utilized RNAi lines from the vdrc KK library underwent diagnostic PCRs according to Green et al. (2014). These validated the hairpin integrations at position 30B on chromosome 2L, and excluded a hairpin integration at position 40D that can be associated with dominant off-target phenotypes (Green et al., 2014; Vissers et al., 2016). S4/actin/28°C KD and phenotypes have previously been published (Foriel et al., 2018) and were reproduced/adapted with the permission from Disease Models and Mechanisms (DMM). In *Drosophila*, NDUFS7 and NDUFV1 have several orthologs (S7: ND-20 and ND-20L; V1: ND-51, ND-51L1, and ND-51L2). We chose to target ND-20 (S7) and ND-51 (V1), which are widely expressed. Since ND-20L (S7), ND-51L1 (V1), and ND-51L2 (V1) presented with high expression solely in the testis and imaginal disc L3, they were not further considered in this study. Throughout *Drosophila* development, four peaks of expression were observed for ND-18, ND-20, and ND-51: (1) a moderate to high expression during the early embryonic stages from embryo 0–2 h to embryo 12–14 h), (2) a very high expression period from late embryogenesis until larval L3 12 h, (3) a moderate to high expression during pupae post white prepupae, and (4) a phase of high/very high expression from mid-pupal stages until 30 days old adults (according to Flybase modENCODE temporal expression data). The actin-Gal4/TM6c, Sb Tb driver was obtained from Christiane Zweier (Humangenetisches Institut, Germany) and w, P{da-Gal4, w+} G32III was obtained from Pascal Heitzler (IGBMC, Strasbourg, France). All stocks were maintained on standard cornmeal-agar medium at 25°C in a 12 h:12 h light dark cycle.

Ten females of the RNAi lines and their appropriate isogenic control line were crossed with 10 males of either the actin- or the da-Gal4 line at 25 or 28°C and 60% humidity. After 24 h of premating, the crosses were transferred to a new vial, referred to as experimental vial, for 2–3 days to raise an adequate population density.

### Eclosion Rate

Fifteen days after the transfer to the experimental vials, the total number of pupae and empty pupae were counted manually with a cell counter for each vial to evaluate the percentage of eclosion. GraphPad Prism 6 (GraphPad software, La Jolla, CA, United States) was used for statistical analysis with Student’s *t*-test with Welch’s correction (when the variance was different) to determine statistical significances (^∗^*P* < 0.05; ^∗∗^*P* < 0.01; ^∗∗∗^*P* < 0.001). Of note, eclosed adult flies of the S4/da/28°C model, frequently fall into the food and die soon after eclosion. As a consequence, the eclosion rate is not necessarily representative of the number of living adult flies. Despite similar eclosion rates between S4/da/28°C and S7/actin/25°C, we could not obtain enough living adult to carry the OxPhos activity measurements. Crosses with daughterless driver and S7 presented with strong delay in pupariation formation.

### OxPhos Enzymatic Activity

One day old adult female KD flies were collected in groups of 30 in triplicates and snap frozen (unless for S7/da/25°C *n* = 1). The 30 flies (whole bodies) were homogenized in 1 ml SETH buffer (0.25 M sucrose, 2 mM EDTA-K, 10 mM Tris, 5 × 104 U/l heparine) with a glass pestle and centrifuged at 600 *g* for 10 min at 4°C. The supernatant was divided in two aliquots of 200 μl and used for OxPhos measurement (complexes I, II, III, and IV). The rest of the supernatant was centrifuged at 14,000 *g* for 10 min at 4°C. The pellet was resolubilized in 400 μl of Tris HCl (pH 7,6) and used for complex V activity measurement. The OxPhos enzyme activities were measured by spectrophotometry with an automated Konelab 20XTi instrument (ThermoFischer) at 37°C. In brief, (1) the rotenone sensitive CI activity was determined following the reduction of 2,6-dichlorophenolindophenol (DCIP) at 600 nm using NADH and coenzyme Q1 as substrate; (2) CII activity was measured as malonate sensitive DCIP reduction at 600 nm using succinate and decylubiquinol as substrates; (3) CIII activity was determined following the reduction of cytochrome c at 550 nm after addition of decylubiquinol and cytochrome c; (4) CIV activity was obtained following cytochrome c oxidation at 550 nm using reduced cytochrome c as a substrate; and finally (5) oligomycin sensitive CV activity was assessed following NADH oxidation at 340 nm using ATP as substrate (see Table 1 in Rodenburg, 2011 for detailed information concentrations, substrates, assay conditions, and readouts). The activity values obtained for each complex were normalized against the citrate synthase (CS) activity and presented as percent of averaged values of the control flies. A multiple comparison test with Bonferroni correction was performed using GraphPad Prism 6 to determine the statistical significance of differences (^∗^*p* < 0.05; ^∗∗^*p* < 0.01; ^∗∗∗^*p* < 0.001).

### Survival

Female KD flies were collected under CO_2_ on the day of eclosion and placed in new vials with fresh food in groups of 20 individuals at the appropriate temperature (25 or 28°C) according to the setting of the experiment. They were transferred to new vials with fresh food every 2–3 days maximum to prevent them to die due to bad food conditions (dry food, mold, or sticky food). The number of dead flies was counted every day. Each experiment was repeated at least two times (unless the eclosion rate did not allow it). Kaplan Meier curves and statistical analysis were generated using the GraphPad Prism 6 survival function, with a log-rank Mantel–Cox test to determine statistical significances (^∗^*p* < 0.05; ^∗∗^*p* < 0.01; ^∗∗∗^*p* < 0.001; ^∗∗∗∗^*p* < 0.0001).

### Quantitative Reverse Transcription – Polymerase Chain Reaction (qRT-PCR)

Primers were designed with the Primer 3 Plus software^1^ using the *D. melanogaster* dNDUFS4 (ND-18, CG12203), dNDUFS7 (ND-20, CG9172), and dNDUFV1 (ND-51, CG9140) nucleic acid sequence from the Ensembl database^2^ (sequences in Table 1).

**Table 1:** Sequences of primers used for qRT-PCR.

Name	Sequence 5′ > 3′
dNDUFS4 forward	AAGATCACCGTGCCGACTG
dNDUFS4 reverse	GACAATGGGTCGCCGCTG
dNDUFS7 forward	GAAGTGGCCCAAAATCTGCC
dNDUFS7 reverse	GAGCAGATCGTCCAGTCTGG
dNDUFV1 forward	TTGGTGGTGAATGCCGATGA
dNDUFV1 reverse	ATGATCTCGCGATCCTTGCA


Prior to experiments, the primer pairs were tested for efficiency (criterion: 0.95 < *R*^2^ < 1) and specificity (criterion: single peak in the dissociation melting curve). To evaluate the RNAi-induced KD efficiency, third instar larvae (*n* = 10 whole larvae per sample) and one day old female adult flies (*n* = 5 whole fly bodies per sample) of the appropriate genotypes (CI subunit crossed with either actin or daughterless driver) and temperature (25 or 28°C) were collected in triplicates and snap frozen in liquid nitrogen. The total RNA from control and KD larvae/adults was isolated following the RNeasy Lipid Tissue Mini kit instructions from Qiagen. The RNA concentration was measured with a nanodrop device (ThermoFischer). DNAse treatment with the DNA-free kit (Ambion) was used to remove genomic contamination prior to cDNA synthesis (Bio-Rad iScript^TM^ Reverse Transcription Supermix). The qPCR was performed using SyBR Green GoTaq^®^ qPCR Master Mix (Promega). Relative gene expression was determined against the geometric mean of two housekeeping genes: γ-tubulin (forward: 5′-TAATGGGCTCGGTCTACTCC-3′ and reverse: 5′-TGTCGAATACCTCTTCTTGCAG-3′) and RNA polymerase 2 (forward: 5′-CCGCGATACTTCTCTCCAC-3′ and reverse: 5′-GACCAGCTAGGCGACATTC-3′). Three biological and two technical replicates were performed for each genotype. Student *t*-test was performed with Welch correction using GraphPad Prism 6 to determine statistical significances (^∗^*p* < 0.05; ^∗∗^*p* < 0.01; ^∗∗∗^*p* < 0.001).

## Results

To develop models for mitochondrial CI deficiencies, we crossed the transgenic RNAi lines to driver lines actin-Gal4/TM6c Sb Tb and da-Gal4, respectively, to induce ubiquitous KD of the CI subunits NDUFS4, NDUFS7, and NDUFV1 (from here on referred to as S4, S7, and V1) at two different temperatures, 25 and 28°C. qRT-PCR analysis at larval and adult stages validated these models and revealed the degree of gene KD in comparison to their appropriate isogenic controls for each subunit of interest in all the tested conditions (three RNAi lines × two driver lines × two temperatures). Overall, all the models presented with a stronger KD at 28°C except for S7/actin and S7/da (Supplementary Figures S1, S2 and Supplementary Table S1). Limited sensitivity of the q-PCR or experimental variation may underlie this exception; no differences were observed between relative S7 expression in the S7/da/25°C and S7/da/28°C KD models (*P* = 0.076), or between their controls (*P* = 0.859). This indicates that the temperature range (25–28°C) does not have effect on S7 transcript levels.

**FIGURE 1:**
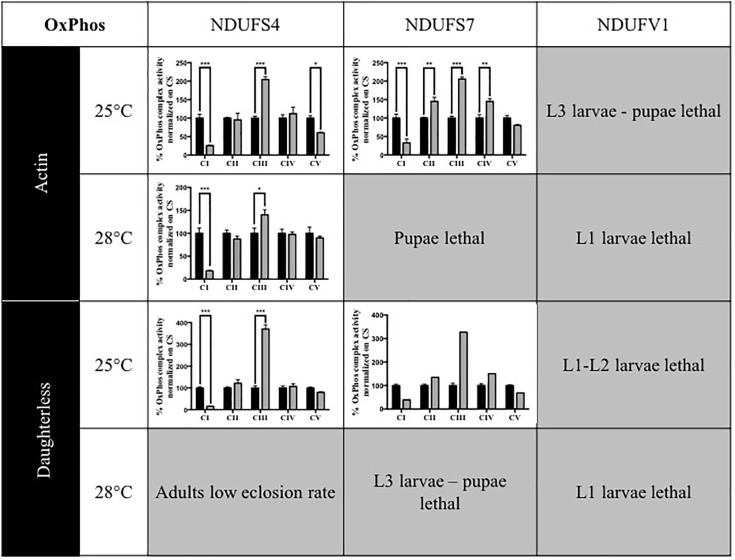
Enzymatic characterization of OxPhos activities in CI KD flies. Enzymatic measurement of the activity of mitochondrial OxPhos complexes, normalized on citrate synthase (CS) activity and presented as a percentage of their respective controls (mean ± SEM, two-way ANOVA, statistical significances: ^∗^*P* < 0.05; ^∗∗^*P* < 0.01; ^∗∗∗^*P* < 0.001). CI activity was significantly reduced in all models. Other complexes activities were either significantly increased (CII in S7/Actin/25°C, CIII in all models, CIV in S7/Actin/25°C and S7/da/25°C) or significantly decreased (CV in S4/Actin/25°C). The numerical values are provided in Supplementary Table S1 (S4/actin/28°C graph reproduced/adapted with permission from Foriel et al., 2018).

In patients, mutations in subunits of complex I can cause isolated or combined OxPhos enzymatic activity disturbances. To determine whether knocking down of *Drosophila* CI subunits leads to comparable defects, we measured the activity of the five OxPhos complexes in the adult KD models and compared these to the complex activities of their isogenic controls and to previous data obtained for the S4 subunit (Foriel et al., 2018). Without any exception, all models that were sufficiently viable to allow collection of adult progenies presented a strong reduction of CI activity. CI activity levels ranged between 15.5 (S4/da/25°C) and 39.3% (S7/da/25°C) of residual activity. In addition, some models showed differences in the activity of other complexes. CIII activity was upregulated in several models, with levels up to 370.4% in the S4/da/25°C model, relative to control levels. S4/actin/25°C was the only model to present a significantly lower CV activity (60.3%), while S7 KD flies instead had an increased CIV activity with both actin and da at 25°C (144.9 and 150.5%) and an increase of CII activity only for S7/actin/25°C (144.8%). The low number of adult escapers available for S7/actin/25°C prevented the generation of replicates and statistical analysis for this specific condition. However, the same tendencies of reduced CI activity and increased CIII activity were observed (Figure 1 and Supplementary Table S1). These data confirm that ubiquitous KD of CI subunits induces CI deficiency in *Drosophila*, in some cases with consequences on the enzymatic activity of other OxPhos complexes.

**FIGURE 2:**
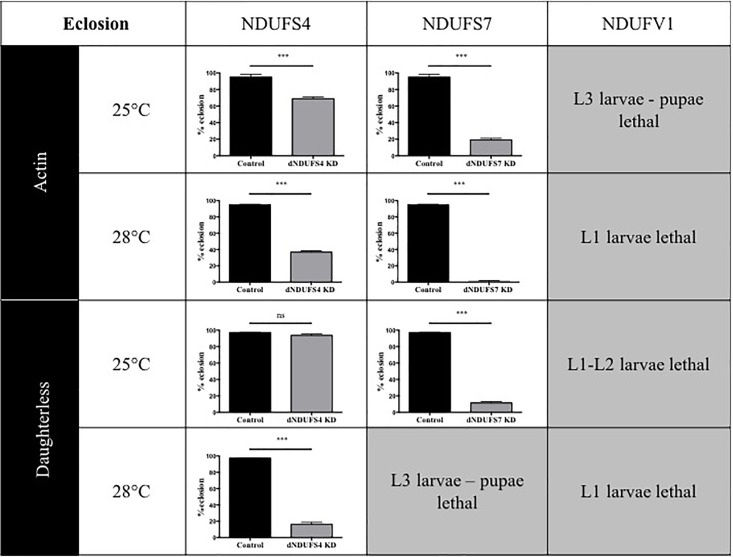
Consequences of CI subunits knockdown for eclosion. Percentage of eclosed pupae from the total number of pupae (mean ± SEM, unpaired Student’s *t*-test, statistical significances: ns, non-significant; ^∗∗∗^*P* < 0.001). Except for S4/da/25°C, all models displayed a reduced eclosion rate as compared to their controls, ranging from 1.6 ± 0.6% in S7/actin/28°C to 68.9 ± 2.2% reduction in S4/actin/25°C. The eclosion rates numerical values are provided in Supplementary Table S1 (S4/actin/28°C graph reproduced/adapted with permission from Foriel et al., 2018).

Having confirmed the CI deficiencies in the panel of generated models, we set out to identify quantitative and disease-relevant phenotypes that can be used as a readout for drug testing. Metamorphosis is a crucial step of *Drosophila* development during which the insects rely solely on the energy reserves acquired during larval development. If the amount of energy stores accumulated is rather low, the animals enter the metamorphosis on a “metabolic edge” which can impact directly on the survival success of the young adult (Merkey et al., 2011). Since defective mitochondria might lead to energy storage defects, we evaluated the percentage of eclosed flies by counting the empty and total number of pupae. Based on our previous work characterizing the S4/actin/28°C model (Foriel et al., 2018), we asked whether eclosion is also affected in S7, V1, and other S4 models. Our collection of CI-deficient models showed a wide range of eclosion rates, which can be classified in four groups (Figure 2): (1) no eclosion impairment such as S4/da/25°C with 93.7% of the flies eclosing compared to 97.1% for its control KK/da/25°C, (2) a modest impairment with S4/actin/25°C and S4/actin/28°C with, respectively, 68.9 and 36.9% of eclosed pupae in comparison to their respective control: control KK/actin/25°C (95.0%) and control KK/actin/28°C (95%), (3) a severe impairment with S4/da/28°C, S7/actin/25°C, S7/da/25°C for which eclosion rates were 16.3, 19.1, and 11.5%, respectively [significant for all of them in comparison to their respective controls: control KK/da/28°C (97.5%), control KK/actin/25°C (95%), and control KK/da/25°C (97.1%)], and (4) very severe eclosion defects with S7/actin/28°C, S7/da/28°C, V1/actin-da/25–28°C, having eclosion rates below 2% and lethality at larval (L1, L2, and L3) and pupal stages. Independently from the subunit targeted, the driver used, or the temperature applied, each model, except S4/da/25°C, showed eclosion defects highlighting a high mortality rate at metamorphosis or pharate stages (Figure 2 and Supplementary Table S1).

**FIGURE 3:**
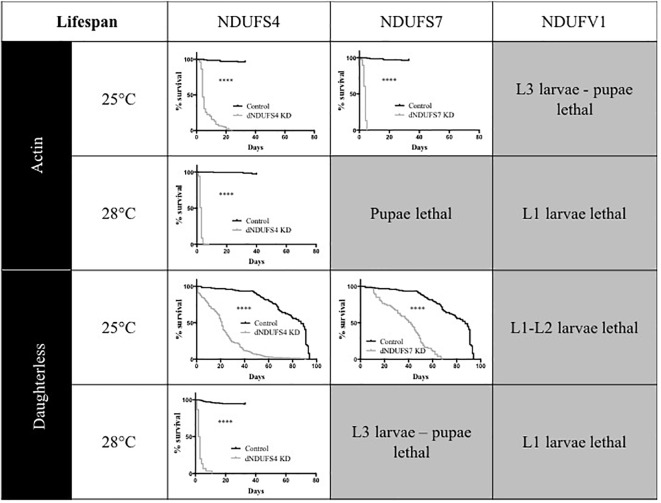
Consequences of CI subunits knockdown on *Drosophila* lifespan. Survival curves of ubiquitous (actin or da) CI subunits KD flies and their corresponding controls. All the models, independently from the drivers and temperatures, presented a significantly shorter lifespan as compared to their respective controls (Kaplan–Meier curves, log-rank Mantel–Cox test, statistical significances: ^∗∗∗∗^*P* < 0.0001). The median survival numerical values are reported in Supplementary Table S1 (S4/actin/28°C graph adapted with permission from Foriel et al., 2018).

One of the hallmarks of early onset CI deficiency consists of premature death at an age below 10 years in most cases (Koene et al., 2012). We therefore investigated adult survival, in addition to the eclosion rates. All *Drosophila* models had a severely reduced lifespan, as illustrated by plotted survival curves (Figure 3) and reduced median survival scores (Supplementary Table S1), the time point (day) at which 50% of adults have died. We distinguished two groups according to severity of survival defects: (1) CI models with a significant and modest lifespan impairment. S4/da/25°C and S7/da/25°C had a median survival of 21 and 42 days, respectively, while their control KK/da/25°C had a median survival of 87 days and (2) CI models with a severe lifespan defect. This group encompassed the S4/actin/25°C, S7/actin/25°C, S4/actin/28°C, and S4/da/28°C models, with median survival scores of 4, 4, 3, and 2.5 days (Figure 3 and Supplementary Table S1). Thus, like patients with mitochondrial disorders, *Drosophila* models with complex I deficiencies show dramatically shortened life expectancy.

## Discussion

Animals are used as models to understand aspects of human physiology and disease pathology that cannot be addressed in patients. In the case of pharmacological development, animal models of diseases play a central role in evaluating the toxicology and efficiency of novel therapeutics. In this study, we generated and described an array of phenotypes for CI deficiencies using *Drosophila* as a model and aimed to identify conditions that could be suitable for future compound testing. Except S7/actin/28°C and S7/da/28°C, all models presented a significant KD of the genes targeted (Supplementary Figures S1, S2) and CI deficiency (Figure 1), validating them as models for mitochondrial CI deficiencies. In addition, the various models showed different ranges of eclosion rate (Figure 2) and survival defects (Figure 3) that could be classified according to the degree of severity. Since S7/actin/28°C and S7/da/28°C did not present a significant reduction of S7 mRNA level (Supplementary Figure S1) and the OxPhos enzymatic activity could not be reliably measured due to limited viability and biological material, these specific conditions are not suited to qualify for S7 deficiency.

Although we focused on a single RNAi line per gene, we have validated the targeted complex I KD, both at the mRNA (qPCR) and the functional level (enzymatic activity). The specificity of our KD phenotypes is supported by (i) the absence of any predicted sequence-related off-target effect of the utilized RNAi constructs/lines (see section “Materials and Methods”), (ii) the exclusion of reported integration site-related off-targets for the KK lines (S4 and S7) (Green et al., 2014; Vissers et al., 2016) (methods, line 90), and (iii) the finding that the observed phenotypes are highly consistent with each other and in accordance with the phenotypes previously described for other CI deficiencies (Cho et al., 2012; Burman et al., 2014; Hedge et al., 2014; Wang et al., 2016). While also the combination of these arguments cannot exclude the occurrence of a minor, undetected off-target effect, they make it extremely unlikely that such off-targets rather than the KD of the CI subunits would account for any of the described phenotypes. It would nonetheless be beneficial to confirm a readout of choice before its usage in a drug screen, e.g., with an independent RNAi line or by a genetic rescue experiment.

It would be also interesting to further characterize these models, e.g., by measuring oxygen consumption, ATP production, and mitochondrial morphology, to obtain more insights into the consequences of the respective genetic and resulting CI deficiencies.

### Comparison of the Models

As expected from the temperature sensitivity of the UAS-Gal4 system, the models raised at 28°C presented with stronger phenotypes than the models raised at 25°C, the only exception being S7/actin/28°C and S7/da/28°C. Actin and daughterless are ubiquitously expressed genes that are subject to dynamic expression changes during the development (according to Flybase data) oscillating between very high and extremely high expression for actin and moderate to very high for daughterless. The actin-Gal4-induced KD models were therefore expected to be more severe, which was the case when looking at adult survival. However, eclosion rates were more affected in da-Gal4-induced models, which suggest that at developmental stages, this driver has a strong component, at least in tissues requiring efficient CI activity. This conclusion is supported by the qPCR data for S4 and S7, either of which at a given temperature shows stronger KD at larval stage upon induction with da-Gal4 and at adult stage with actin-Gal4. The adult survival, at least considering S4 for which all conditions could be assessed, also grossly correlated with this notion and with adult KD efficiency (Actin-Gal4/28°C being to the most stringent and da-Gal4/25°C being the most permissive condition).

Intriguingly, we observed a consistent increase of CIII activity in all the models measured. Patients with CI deficiency can show either isolated CI deficiency or multiple OxPhos complex deficiencies, the latter of which may include reduced CIII activity (Budde et al., 2000; Ulgade et al., 2004). Significantly increased CIII activity has not been reported in our clinic. Although we cannot exclude that the observation of a significant CIII activity increase is favored in *Drosophila* versus humans by less variation due to the isogenic genetic background, our observation may also reflect an organism-specific mechanism of the OxPhos system or its converging pathways. Further experiments such as oxygen consumption measurements to further understanding potentially diverging mechanisms, and validation of any identified drug in other systems is indispensable. In this study, we focus on CI, and despite quantitative differences in eclosion rate and enzyme activity, CI deficiency was efficiently induced.

### Utility of the Models for Drug Screening: Quantity Versus Quality

The use of different Gal4 lines and variation of culture temperatures allowed us to create a panel of models with varying severity. This, to some degree, mirrors the broad range of severity seen in patients with defects in NDUFS4, S7, and V1, and in mitochondrial disorders in general. This panel of models could allow the evaluation of drugs on phenotypes of different strength. Which one(s) to select for drug screening is an important question and can be expected to represent a trade-off between throughput and sensitivity. Screening for rescue of severe phenotypes such as developmental lethality/eclosion defects can be performed, at least with an appropriate setup, in high throughput as demonstrated by Chang et al. (2008), for the *Drosophila* model of Fragile X syndrome, but obtaining rescue may be challenging and require highly effective drugs.

Phenotypes belonging to both ends of the spectrum have been identified by this study. Very severe phenotypes with fully penetrant lethality in early developmental stages would allow quantitative screening for survivors upon drugs treatment, e.g., in V1/actin/25°C. Milder phenotypes on the other hand might be more sensitive and therefore easier to rescue but are probably less convenient in terms of time and feasibility for the screening of compound libraries. The reduced lifespan determined for S4 or S7 upon induction with actin at 25°C, S4/actin/28°C as well as S4 or S7/da/25°C appear to represent such conditions. Ideally, one would go for both approaches, if possible.

### Alternative Approaches

Taking advantage of the UAS-Gal4 system, the presented phenotypes could be modulated further, e.g., by further increasing or decreasing the temperature, by using driver lines containing a copy of UAS-Dicer-2, or by using tissue-specific instead of ubiquitous Gal4 lines. A principle alternative to our approach lies in the generation of stable genetic mutants. With the expansion of CRISPR/Cas9 gene editing technologies, the possibilities to target each gene of interest have grown, opening new perspectives. A CRISPR/Cas9 mutant of the phosphomannomutase 2 (a disorder of glycosylation) presenting lethality in larval stages has, for example, been used to perform high throughput screening of compounds in 96 well plates^3^. Once a mutant (point mutation, null mutant) with a suitable phenotype is identified, this surely represents a valuable and powerful tool, which may even have less variability in phenotype. However, the challenge lies in obtaining a mutant with such a suitable phenotype, since that system does not allow any fine-tuning and lacks flexibility for phenotype adjustments.

Ultimately, fly models could potentially be used instead of cell-based screening, or be utilized in parallel to cellular platforms, in order to avoid discarding potential lead compounds that fail to arise from *in vitro* studies, e.g., because they rely on a systemic mechanism. In summary, this study and catalog of phenotypes presented here offer relevant tools and starting points that can be exploited to dissect the pathomechanism of CI deficiencies and for the development of pharmaceutics through high-throughput and/or sensitive approaches.

## Data Availability

Datasets are available on request: The raw data supporting the conclusions of this manuscript will be made available by the authors, without undue reservation, to any qualified researcher.

## Ethics Statement

In this study, we used *Drosophila*, an invertebrate, that according to European legislation is not an animal. No ethical approvals are required.

## Author Contributions

SF, GR, YL, JoB, and RR performed the experiments. SF, YL, and JoB analyzed the data. SF, JuB, AS, and JS designed the research and wrote the manuscript. JuB, AS, and JS were responsible for project administration.

## Conflict of Interest Statement

JS the founding CEO and JuB the COO of Khondrion B.V., SF and JoB were employed by Khondrion B.V. The remaining authors declare that the research was conducted in the absence of any commercial or financial relationships that could be construed as a potential conflict of interest.

## Footnotes

1 http://www.bioinformatics.nl/cgi-bin/primer3plus/primer3plus.cgi/

2 http://www.ensembl.org/index.html

3 https://www.perlara.com

## Supplementary Material

FIGURE S1 Validation of the CI subunit knockdown by qRT-PCR in larvae.

FIGURE S2 Validation of the CI subunit knockdown by qRT-PCR in adults flies. Transcript level of the different targeted subunits of complex I presented as percentage of their respective isogenic background controls (mean ± SEM, unpaired Student’s *t*-test, statistical significances: ns, non-significant; ^∗^*P* < 0.05; ^∗∗^*P* < 0.01; ^∗∗∗^*P* < 0.001). The numerical values are reported in Supplementary Table S1 (qRT-PCR graph for adult flies S4/actin/28°C reproduced/adapted with permission from Foriel et al., 2018).

TABLE S1 Numerical values for each phenotype quantified in the different models: qRT-PCR in larvae, qRT-PCR in adults, OxPhos measurement, eclosion rate, survival (median survival).
