# Supplementary figures and images for: A Drosophila Mitochondrial Complex I Deficiency Phenotype Array
Source: Front Genet. 2019 Mar 27;10:245. doi: 10.3389/fgene.2019.00245 (PMC6445954; doi:10.3389/fgene.2019.00245)

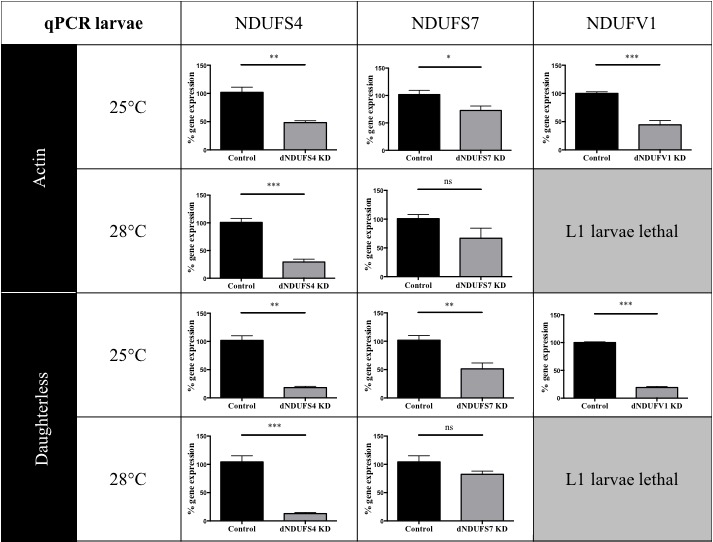

Supplement: FIGURE S1 — Validation of the CI subunit knockdown by qRT-PCR in larvae. [file Image_1.JPEG]

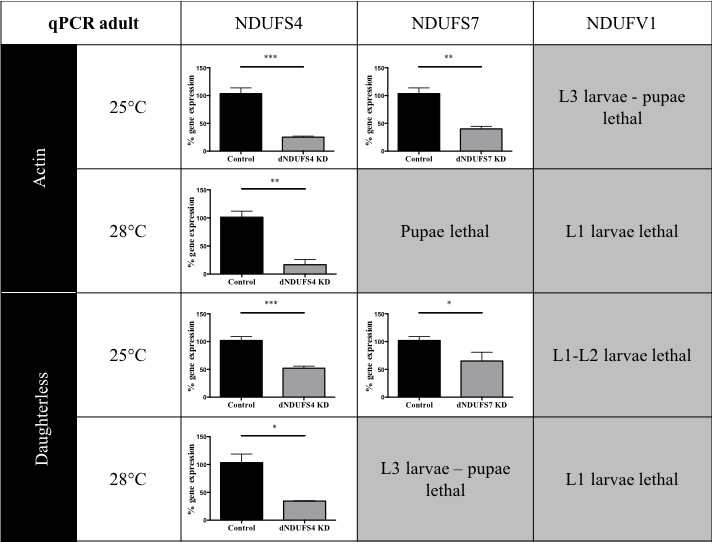

Supplement: FIGURE S2 — Validation of the CI subunit knockdown by qRT-PCR in adults flies. Transcript level of the different targeted subunits of complex I presented as percentage of their respective isogenic background controls (mean ± SEM, unpaired Student’s t-test, statistical significances: ns, non-significant; ∗P < 0.05; ∗∗P < 0.01; ∗∗∗P < 0.001). The numerical values are reported in Supplementary Table S1 (qRT-PCR graph for adult flies S4/actin/28°C reproduced/adapted with permission from Foriel et al., 2018). [file Image_2.JPEG]
